# Prevalence and sociogeographical inequalities of violence against women in Ecuador: a cross-sectional study

**DOI:** 10.1186/s12939-021-01456-9

**Published:** 2021-06-02

**Authors:** Agnes Edeby, Miguel San Sebastián

**Affiliations:** Department of Epidemiology and Global Health, Umeå University, Umeå, Sweden

**Keywords:** Violence, Women, Prevalence, Risk difference, Ecuador

## Abstract

**Background:**

Violence against women (VAW) is a vast public health problem in Latin America. The aim of this study was to determine the prevalence of violence against women and to assess its sociogeographical inequalities in Ecuador during 2019.

**Methods:**

This cross-sectional study used data from a national survey conducted in 2019 (*n* = 17,211) among women aged 15 years and over. Independent variables included age, marital status, education, ethnicity, place of residence and region. The chosen outcomes were lifetime experience of total violence, sexual violence, physical violence, psychological violence and economic violence. Frequency tables were first calculated, and then crude and adjusted regression models estimating risk differences and their 95% confidence intervals were computed.

**Results:**

Nearly two-thirds **(**64.86%) of the participating women had experienced some form of violence during their lifetime, mainly psychological violence (56.92%). The second most prevalent type of violence was physical (35.44%) closely followed by sexual (32.67%). Almost one-fifth (16.38%) stated to have experienced some form of economic violence.

Physical and psychological violence were more common among women aged 26–35 and less among older women. All forms of violence were more often reported among women with no education except for sexual violence, which was more frequent among those with higher education. Physical, psychological and economic violence were more often reported by those living with a partner, being married or divorced/separated. Sexual violence was associated with those belonging to a non-indigenous ethnic group, while all types of violence were more common among those living in an urban setting (except physical violence), in the Highlands or in the Amazon region.

**Conclusions:**

Our study showed that VAW is a common event in Ecuador and identified several sociogeographical inequalities that varied depending on the type of violence. VAW was more common among the younger age groups, those with low education, those living with a partner or being divorced/separated, or residing in an urban setting, in the Highlands or in the Amazon. Further studies including more social factors and a continuous monitoring of VAW are recommended. Current policies and laws to protect women need to be expanded and effectively implemented to reduce VAW in the Ecuador.

**Supplementary Information:**

The online version contains supplementary material available at 10.1186/s12939-021-01456-9.

## Introduction

Violence Against Women (VAW) is commonly defined as “any act of gender-based violence that results in, or is likely to result in, physical, sexual or psychological harm or suffering to women, including threats of such acts, coercion or arbitrary deprivation of liberty, whether occurring in public or in private life.” [1] The term is rooted in sexism, patriarchy and gender inequality and as such is pervasively exercised against women in three interconnected spheres: family, community and the state [2]. Research has demonstrated that VAW is a major cause of ill health among women and girls, whose impact can be seen directly through death and disability due to injuries, as well as indirectly through increased vulnerability to a host of physical and mental health problems [3–5].

Globally, 35.6% of women have ever experienced either non-partner sexual violence or physical or sexual violence by an intimate partner, or both, with intimate partner violence (IPV) being the most common form [5]. Worldwide, almost one-third (30%) of all women who have been in a relationship have experienced physical and/or sexual violence by their intimate partner [5]. Variations between countries or regions are extensive, however. While the prevalence of lifetime VAW is highest in the World Health Organization (WHO) African (45.6%) and South East Asian (40.2%) regions, the situation in the Americas region is also of concern (36.1%) [5]. Despite the recognized extension of the problem, studies focused on both partner and non-partner violence in Latin America are difficult to find. Due to this, a recent WHO review comparing violence against women in 12 Latin American countries concentrated only on IPV. All countries had a widespread prevalence for IPV (physical and sexual) among women ever married or in a relationship, results varying between the ranges of 17 to 53.3% [6]. Only four of the countries included questions regarding non-partner violence in their population-based surveys. For instance, in Haiti 26.5% of women older than 15 years old had experienced physical violence by any perpetrator during their lifetime [7]. In the Dominican Republic, 20 and 10% of women over 15 years reported physical and sexual violence ever in their lifetime, respectively [8]; and in Peru, 39.5 and 19.7% of women referred to ever having experienced physical violence by a partner and a non-partner, respectively [9].

The social ecological framework is a useful tool to describe and categorize different risk factors for becoming exposed to violence. Originally described by Heise in 1998, adaptations to the framework classify the risk factors into four levels: individual, relationship, community and societal. Some common factors associated with VAW at the different levels include being abused as a child, use of alcohol, male dominance within a relationship, low socioeconomic status, poverty, rigid gender roles and cultural norms that accept interpersonal violence [10, 11].

While specific risk factors for VAW such as armed conflicts, criminal violence and a male dominance culture (patriarchy, machismo) have been identified for the Latin American context [11, 12], the literature regarding risk factors for being exposed to VAW, or even IPV, is scarce.

A study from Brazil in 2010 identified low level of education, mental illness and a partner that consumes alcohol as main factors for violence among women visiting health care services [13]. In addition to those factors, urban residence and women being employed were reported as significant risk factors for physical, psychological and sexual violence by an intimate partner in a study from Bolivia in 2012 [14].

Ecuador has made a great effort, particularly in the last two decades, to create a legal framework to prevent, eliminate and punish all forms of VAW. In addition to the ratification of international conventions [15], in 2014 Ecuador adopted the “Organic Law on the Integrated Criminal Code” (COIP), which provided definitions of violence and specific penalties for VAW. In 2018 the country went further and approved the Comprehensive Organic Law to Prevent and Eradicate Violence against Women [16] which includes three components for the eradication of violence: attention, protection and reparation of women victims of violence to guarantee their safety and integrity and to resume their life project.

The first National Survey of Family Relations and Gender Violence against Women in Ecuador was performed by the Ecuadorian National Institute of Statistics and Census (INEC in Spanish) in 2011 and included 18,800 women aged 15 years old or above [17]. Results showed that 60% of women had experienced some form of VAW (including psychological, physical, sexual and economic violence), psychological violence (53.9%) being the most common type of violence. In women that had experienced physical violence, it was most typically carried out by an intimate partner [17].

In 2019, the INEC carried out a second survey, not only to respond to the obligation of collecting gender-based violence information assigned by the Institute in the 2018 Law but also to provide updated evidence for the indicators of the National Development Plan and the 2030 Agenda for Sustainable Development related to gender violence [18]. While descriptive results of this survey have been published [18], a systematic analysis of risk factors for VAW has not. The aim of this study was therefore to determine the prevalence of VAW and to assess the sociogeographical inequalities among the different types of violence in Ecuador.

## Methods

### Setting

Ecuador, located in the northwest part of South America, had an estimated population of 16.9 million people in 2020 [19] and is considered a middle-income country [20]. The country is divided into four regions: three on the mainland - the Coast, the Highlands and the Amazon - and the Galapagos Islands [21]. The main export commodities include oil-related products and bananas [19]. Several ethnic groups exist, being mestizo (71.9%, a mix of Amerindian and white), followed by the Montubio (7.4%, a mestizo group in the coastal region), the largest ones; some other groups include the Indigenous nationalities (7%) and the Afro-Ecuadorians (4.3%) [19]..

### Study design

This study followed a cross-sectional design based on the second National Survey of Family Relations and Gender Violence against Women in Ecuador conducted by the INEC in 2019. The survey aimed to assess the prevalence of different types of violence against women aged 15 years and older. A national representative sample of 17,211 women participated in the survey, and the data are publicly available [22].

A three-stage cluster sampling design was used to obtain national and regional as well as rural/urban estimates of the population. In the first stage, primary sampling units were selected and 8 households chosen in each of them. Finally, the Kish table was used to select one woman per household. The sample size was calculated based on previous estimates of the prevalence of violence nationally [17], using a confidence interval of 5% and a non-response rate of 5% resulting in the selection of 25,427 households including 27,842 women (15 years and older). Because only one woman per household was randomly selected, a total of 19,161 women formed the target population. Due to lack of response (not wanting to participate or absence from home), 17,211 (89.82%) women constituted the final study population. Detailed methodological procedures of the survey have previously been described [23].

### Measures

#### Dependent variables

Four dimensions of violence were explored in the survey: psychological, sexual, physical and economic with several questions included in each dimension. Gynaecological and obstetric violence information was also collected but not included in this study. These VAW-related questions were prepared based on the guidelines of the National Plan for Eradication of Gender Violence [23, 24]. All questions for this study refer to reported experiences of violence at least once during the participants´ lifetime.

Physical violence was characterized as physical aggression or use of weapons or objects to cause injury; psychological violence as threatening behaviours, humiliation or insult; sexual violence as sexual relations imposed by physical force or threats and any imposed acts that were considered humiliating; and economic violence as the damage, theft, destruction or retention of goods, values, rights or economic resources destined to satisfy the needs of the victims. These questions were asked in relation to two main environments: the public sphere (school, work and social) and private sphere (family and partner relationships). Total violence was the sum of exposure to the four dimensions of violence.

Women who answered “Yes” to at least one of the questions that referred to each type of violence were considered exposed to violence.

#### Independent variables

The independent variables were chosen based on the availability of data and organized using the social-ecological model into individual, relationship and societal factors [11]. Two variables from the survey were considered as individual factors. Age was grouped into six categories: 15–25, 26–35, 36–45, 46–55, 56–65 and >  65; while education was grouped into four: no education, primary/middle, secondary and university or higher. The survey included a question on financial aid, but only 1437 women answered the question. Therefore, this variable was excluded from the analysis. Marital status (relationship factor) was recoded into single, living with partner, married, separated/divorced and widowed. Ethnicity and place of residence were included as societal factors. Ethnicity was recoded into Indigenous, people of colour (POC) (Afro-Ecuadorian, mulatto, Montubio), mestizo and white and other. Since ethnicity includes a strong cultural dimension, it was categorized as a societal factor. Geography was captured with two variables: area of residence, which was grouped into rural and urban; and regional, divided into four categories: the Coast, the Highlands, the Amazon, and the Galapagos and others (undefined areas).

### Statistical analysis

Frequency tables and percentages were used to present the descriptive characteristics of the population and the health outcomes according to the different types of violence (see also appendix 1 for within-group comparisons).

Bivariate analyses between the independent variables and the violence outcomes were carried out first. All covariates that were statistically significant were then included in a multivariable regression model. Risk differences (RD) and their 95% confidence intervals (95% CI) were calculated as the measure of association.

Weighting factors were calculated by the statisticians responsible of the national survey based on the inverse probability of being selected in each of the previously mentioned sampling stages. Sample weighting was applied in all analyses to adjust for the unequal probability of sample selection and interview. A detailed explanation of the weighting procedure can be found in the Survey Manual [25]. The Huber/Whites/sandwich estimator was also applied to obtain robust standard errors. In addition, collinearity was checked, with all the independent variables having a variance inflation factor (VIF) lower than 1.5. The Stata 14 software was used to conduct the analyses.

### Ethical consideration

All women that participated in the study gave their consent. Since the data is publicly available, no ethical approval was necessary to conduct this study.

## Results

### Description of the study sample

Overall, 17,211 women participated in the study, the majority being between 15 and 25 years old (24.48%), followed by the 36–45 age group (17.71%). The majority had finished secondary education (40.65%) followed by those with primary/middle education (33.11%). Regarding marital status, most were married (32.10%) and single (26.47%). Being part of the Mestizo ethnic group was by far the most common (82.13%), and most participants lived in urban areas (71.32%). Of the four different regions, the majority of participants lived in the Highlands and Coast (46.39 and 48.70%, respectively) (Table 1).

**Table 1:** Characteristics of study participants (as column percentages) and prevalence of the different outcomes in total and by sociogeographical factors (as row percentages), Ecuador 2019.

Variables		Total sample (%)	Total violence (%)	Sexual violence (%)	Physical violence (%)	Psychological violence (%)	Economic violence (%)
	**Total sample**	17,211 (100)	11,163 (64.86)	5622 (32.67)	6100 (35.44)	9797 (56.92)	2819 (16.38)
**Individual**							
**Age**	15–25	4213 (24.48)	2431 (57.73)	1608 (38.17)	919 (21.80)	1853 (43.99)	362 (8.60)
	26–35	2816 (16.36)	1985 (70.50)	1117 (39.67)	1108 (39.35)	1741 (61.82)	493 (17.51)
	36–45	3048 (17.71)	2071 (67.96)	961 (31.52)	1142 (37.48)	1885 (61.85)	596 (19.54)
	46–55	2813 (16.34)	1856 (66.00)	881 (31.34)	1147 (40.79)	1696 (60.32)	547 (19.46)
	56–65	2270 (13.19)	1467 (64.63)	594 (26.18)	953 (41.97)	1358 (59.81)	422 (18.59)
	> 65	2051 (11.92)	1351 (65.84)	461 (22.44)	832 (40.53)	1264 (61.60)	399 (19.46)
**Education**	University or higher	3631 (21.10)	2270 (62.52)	1498 (41.26)	984 (27.11)	1852 (51.00)	492 (13.56)
	Secondary	6997 (40.65)	4536 (64.83)	2543 (36.34)	2317 (33.12)	3896 (55.69)	1110 (15.87)
	Primary/Middle	5699 (33.11)	3733 (65.51)	1411 (24.76)	2340 (41.07)	3455 (60.62)	1020 (17.90)
	None	885 (5.14)	624 (70.50)	170 (19.27)	459 (51.84)	595 (67.21)	197 (22.26)
**Relationship**							
**Marital status**	Single	4555 (26.47)	2502 (54.93)	1687 (37.05)	903 (19.83)	1866 (40.96)	250 (5.48)
	Living with partner	3562 (20.70)	2424 (68.05)	1119 (31.42)	1396 (39.18)	2208 (61.97)	527 (14.80)
	Married	5524 (32.10)	3494 (63.25)	1473 (26.66)	1937 (35.06)	3170 (57.39)	664 (12.02)
	Separated/divorced	2474 (14.37)	2073 (83.82)	1118 (45.19)	1397 (56.48)	1020 (77.60)	1163 (47.03)
	Widowed	1097 (6.37)	670 (61.05)	225 (20.53)	468 (42.66)	634 (57.84)	216 (19.65)
**Societal**							
**Ethnicity**	Indigenous	1237 (7.19)	793 (64.05)	244 (19.71)	550 (44.42)	728 (58.84)	201 (16.26)
	POC	1204 (7.00)	780 (64.80)	389 (32.35)	450 (37.40)	711 (59.06)	216 (17.94)
	Mestizo	14,135 (82.13)	9199 (65.08)	4741 (33.54)	4905 (34.70)	8022 (56.75)	2318 (16.40)
	White and others	635 (3.69)	391 (61.60)	248 (39.05)	196 (30.79)	336 (56.92)	85 (13.36)
**Area**	Rural	4936 (28.68)	3098 (62.77)	1131 (22.91)	1883 (38.16)	2832 (57.38)	733 (14.86)
	Urban	12,275 (71.32)	8065 (65.70)	4492 (36.59)	4217 (34.35)	6965 (56.74)	2086 (16.99)
**Regions**	Coast	8382 (48.70)	5022 (59.91)	2620 (31.26)	2603 (31.05)	4374 (52.19)	1226 (14.63)
	Highlands	7984 (46.39)	5555 (69.57)	2757 (34.53)	3132 (39.22)	4887 (61.21)	1448 (18.14)
	Amazon	789 (4.58)	556 (70.50)	233 (29.52)	349 (44.27)	510 (64.60)	141 (17.86)
	Galapagos and others	56 (0.33)	30 (53.33)	13 (22.90)	17 (35.44)	26 (46.24)	5 (8.12)

Table 1 presents the prevalence of the different types of VAW in total and by sociogeographical variables. Of all women, 11,163 (64.86%) reported having experienced some kind of violence during their lifetime, mainly psychological violence (56.92%). The highest prevalence of any kind of violence was in the age group 26–35 years old (70.50%). Regarding other forms of violence, some patterns emerged; reported sexual violence was more common among young women (39.67% in the age group 26–35 years old) while older women (49.97% in the age group 56–65 years old) reported more physical violence. For psychological and economic violence, the prevalence was similarly distributed among the different age groups.

Women who had not been able to attend school reported the highest prevalence of total violence (70.50%) and of all other types except for sexual violence, which was highest (62.52%) among women who had a university degree or higher education.

Concerning marital status, women separated/divorced reported the highest prevalence in total violence (83.82%) as well as in the other forms of violence.

Regarding ethnicity, the prevalence of total violence was quite similar between the different groups. The reporting of sexual violence was most common among the ethnic group white and others (39.05%), physical violence among the Indigenous group (44.42%), and psychological and economic violence among the ethnic group POC (59.06 and 19.94% respectively).

While the prevalence of violence was quite similar between urban and rural areas, in terms of regions, the highest prevalence of total violence was in the Highlands and Amazon regions (69.57 and 70.50%, respectively). Sexual violence was highly reported in all three regions on the mainland but most commonly in the Highlands region (34.53%) where economic violence also had the highest prevalence (18.14%). However, physical and psychological violence were more common in the Amazon region (44.27 and 64.60%).

Frequencies for IPV were also calculated (numbers not included in tables) and results showed a prevalence of 44.81% for psychological violence, 28.49% for physical violence, 8.51% for sexual violence and 14.41% for economic violence.

### Regression analyses

All independent variables were statistically significant associated to all the different types of violence except ethnicity for total, psychological and economic violence in the crude analyses (Table 2).

**Table 2:** Weighted crude risk differences and their 95% confidence intervals (95% CI) between the independent variables and the violence outcomes, Ecuador 2019.

Variables		Total violence	Sexual violence	Physical Violence	Psychological violence	Economic violence
**Individual**						
**Age**	15–25	0	0	0	0	0
	26–35	12.77 (9.01, 16.53)	1.50 (−2.37, 5.38)	17.54 (14.03, 21.06)	17.82 (13.93, 21.72)	8.90 (6.31, 11.49)
	36–45	10.23 (6.40, 14.06)	−6.65 (−10.39, − 2.91)	15.67 (12.21, 19.14)	17.86 (13.96, 21.76)	10.94 (8.21, 13.66)
	46–55	8.28 (4.24, 12.31)	−6.83 (− 10.79, − 2.86)	19.0 (15.2, 22.7)	16.33 (12.22, 20.43)	10.85 (8.01, 13.69)
	56–65	6.90 (2.73, 11.08)	−11.99 (− 16.01, −7.96)	20.16 (16.21, 24.11)	15.82 (11.57, 20.06)	9.99 (7.01, 12.96)
	> 65	8.12 (3.99, 12.25)	−15.72 (− 19.71, − 11.74)	18.73 (14.86, 22.59)	17.61 (13.40, 21.82)	10.86 (7.90, 13.82)
**Education**	University or higher	0	0	0	0	0
	Secondary	2.31 (−1.08, 5.70)	−4.92 (−8.32, − 1.51)	6.02 (2.93, 9.10)	4.69 (1.22, 8.15)	2.31 (− 0.10, 4.71)
	Primary/Middle	3.0 (− 0.42, 6.42)	−16.50 (− 19.78, − 13.22)	13.96 (10.82, 17.10)	9.63 (6.16, 13.10)	4.35 (1.94, 6.75)
	None	7.98 (3.16, 12.80)	−21.99 (−26.66, − 17.33)	24.73 (19.55, 29.91)	16.21 (11.22, 21.21)	8.70 (4.23, 13.11)
**Relationship**						
**Marital Status**	Single	0	0	0	0	0
	Living with partner	13.13 (9.54, 16.71)	−5.62 (−9.13, − 2.11)	19.35 (16.11, 22.59)	21.01 (17.39, 24.63)	9.32 (7.18, 11.46)
	Married	8.33 (5.01, 11.64)	−10 .38 (−13.6, −7.21)	15.24 (12.41, 18.06)	16.42 (13.12, 19.73)	6.54 (4.84, 8.24)
	Separated/divorced	28.89 (25.24, 32.54)	8.14 (3.84, 12.44)	36.65 (32.64, 40.66)	36.64 (32.71, 40.57)	41.54 (37.95, 45.14)
	Widowed	6.13 (0.86, 11.39)	−16.52 (−20.88, − 12.15)	22.83 (18.01, 27.65)	16.87 (11.63, 22.12)	14.17 (10.50, 17.84)
**Societal**						
**Ethnicity**	Indigenous	0	0	0	0	0
	POC	0.75 (−4.93, 6.44)	12.63 (7.45, 17.82)	−7.02 (− 12.64, −1.40)	0.22 (−5.59, 6.02)	1.67 (−2.78, 6.12)
	Mestizo	1.03 (−2.87, 4.93)	13.83 (10.63, 17.02)	−9.72 (−13.52, −5.91)	− 2.09 (− 6.04, 1.86)	0.13 (− 2.78, 3.05)
	White and others	−2.45 (− 10.40, 5.51)	19.34 (11.62, 27.06)	−13.63 (− 21.10, − 6.16)	−5.94 (− 14.02, 2.14)	−2.90 (−8.08, 2.28)
**Area**	Rural	0	0	0	0	0
	Urban	2.93 (0.44, 5.42)	13.69 (11.39, 15.98)	−3.80 (−6.21, −1.40)	−0.64 (− 3.20, 1.91)	2.14 (0.35, 3.92)
**Regions**	Coast	0	0	0	0	0
	Highlands	9.66 (7.20, 12.13)	3.27 (0.82, 5.72)	8.18 (5.78, 10.58)	9.03 (6.47, 11.58)	3.51 (1.64, 5.39)
	Amazon	10.59 (7.92, 13.26)	−1.74 (−4.33, 0.85)	13.22 (10.54, 15.90)	12.41 (9.65, 15.17)	3.24 (1.13, 5.34)
	Galapagos and others	−6.59 (− 14.0, 0.83)	−8.35 (− 14.50, −2.20)	−0.54 (− 7.06, 5.98)	−5.94 (− 13.22, 1.34)	−6.51 (−9.79, − 3.22)

Table 3 presents the results of the multivariable regression analyses. After adjusting, the adjusted risk difference (ARD) for total violence was significantly smaller in the oldest (> 65) age group (ARD: -4.99; 95% CI: − 9.90, − 0.08). However, the pattern changed according to the type of violence. While sexual and economic violence were also lower among older groups, physical (ARD: 10.92; 95% CI: 7.09, 14.75) and psychological violence (ARD: 9.34; 95% CI: 5.17, 13.52) were more common in the 26–35 years old age group.

**Table 3:** Weighted adjusted risk differences and their 95% confidence intervals (95% CI) between the independent variables and the violence outcomes, Ecuador 2019.

Variables		Total violence	Sexual violence	Physical Violence	Psychological violence	Economic violence
**Individual**						
**Age**	15–25	0	0	0	0	0
	26–35	6.19 (1.99, 10.40)	0.02 (−4.15, 4.20)	10.72 (6.91, 14.54)	8.97 (4.83, 13.12)	2.76 (0.15, 5.36)
	36–45	1.82 (−2.75, 6.40)	−6.42 (−10.59, − 2.25)	5.62 (1.49, 9.75)	7.17 (2.78, 11.55)	2.70 (−0.19, 5.59)
	46–55	0.26 (−4.53, 5.05)	− 6.03 (− 10.38, − 1.67)	8.93 (4.64, 13.21)	5.58 (1.00, 10.16)	2.28 (− 0.79, 5.34)
	56–65	−1.44 (− 6.59, 3.72)	−9.59 (− 14.11, −5.08)	8.36 (3.73, 12.98)	4.25 (− 0.58, 9.09)	0.78 (− 2.62, 4.17)
	> 65	0.44 (− 5.04, 5.91)	−11.38 (− 16.14, − 6.62)	4.41 (−0.59, 9.40)	5.31 (0.04, 10.58)	0.14 (− 3.59, 3.88)
**Education**	University or higher	0	0	0	0	0
	Secondary	4.28 (0.66, 7.90)	−3.74 (−7.19, −0.30)	7.89 (4.91, 10.86)	6.73 (3.41, 10.05)	3.91 (1.67, 6.15)
	Primary/Middle	3.65 (−0.21, 7.52)	−9.32 (−12.90, −5.74)	11.56 (8.25, 14.87)	8.74 (5.22, 12.27)	4.27 (1.83, 6.72)
	None	10.74 (5.02 16.45)	−8.61 (−13.74, −3.47)	20.53 (14.61, 26.44)	13.61 (8.14, 19.07)	8.21 (3.34, 13.09)
**Relationship**						
**Marital Status**	Single	0	0	0	0	0
	Living with partner	15.28 (11.06, 19.50)	0.89 (−2.46, 5.06)	15.34 (11.66, 19.02)	19.12 (15.15, 23.09)	9.04 (6.75, 11.34)
	Married	7.87 (3.70, 12.04)	−2.44 (−5.28, 2.00)	7.61 (4.04, 11.18)	11.09 (7.12, 15.06)	4.70 (2.49, 6.92)
	Separated/divorced	29.86 (25.50, 34.21)	13.92 (5.33, 13.65)	31.89 (27.39, 36.39)	33.35 (29.02, 37.69)	40.73 (36.94, 44.52)
	Widowed	6.89 (0.60, 13.19)	−1.04 (−5.92, 3.82)	14.82 (9.01, 20.56)	12.08 (6.06, 18.10)	13.12 (8.78, 17.47)
**Societal**						
**Ethnicity**	Indigenous	–	0	0	–	–
	POC	–	10.15 (4.62, 15.68)	−0.62 (−6.68, 5.43)	–	–
	Mestizo	–	7.85 (4.44, 11.26)	−2.09 (− 6.39, 2.21)	–	–
	White and others	–	14.39 (6.77, 22.00)	−3.85 (−10.61, 2.92)	–	–
**Area**	Rural	0	0	0	0	0
	Urban	5.85 (2.98, 8.72)	8.89 (6.38, 11.40)	1.69 (−0.99, 4.37)	3.19 (0.53, 5.84)	2.32 (0.49, 4.16)
**Regions**	Coast	0	0	0	0	0
	Highlands	13.38 (10.70, 16.06)	5.69 (3.24, 8.14)	11.08 (8.60, 13.56)	12.07 (9.53, 14.60)	6.30 (4.53, 8.08)
	Amazon	13.37 (10.38, 16.36)	5.13 (2.31, 7.96)	13.49 (10.51, 16.47)	13.66 (10.92, 16.40)	5.06 (3.07, 7.05)
	Galapagos and others	−3.89 (−11.41, 3.63)	−1.43 (−7.94, 5.08)	−0.43 (−6.25, 5.39)	−5.06 (− 12.27, 2.15)	−4.76 (−8.17, − 1.36)

An increased risk difference in total violence (ARD: 7.39; 95% CI: 2.26, 12.52), physical (ARD: 18.67; 95% CI: 12.78, 24.54), psychological (ARD: 11.10; 95% CI: 5.64, 16.561) and economic violence (ARD: 5.42; 95% CI: 0.49–10.35) was observed among women who had not been able to attend school. In contrast, women with a university level education or higher reported sexual violence more often when compared to those with no educational degree (ARD: 12.34; 95% CI: 7.26–17.42, results not included in Table 3).

Living with a partner (ARD: 15.28; 95% CI: 11.06, 19.50), being married (ARD: 7.87; 95% CI: 3.70, 12.04), separated/divorced (ARD: 29.86, 95% CI: 25.50, 34.21) and widowed (6.89; 95% CI: 0.60, 13.19) remained statistically associated with total violence. Other significant findings regarding marital status were that being separated/divorced resulted in a considerable risk difference for all the separate forms of violence, the largest for economic violence (ARD: 40.73; 95% CI: 36.94, 44.52) and the second largest for psychological violence (ARD: 33.35; 95% CI: 29.02, 37.69).

Sexual violence was more often reported among all non-Indigenous (white and others, ARD: 14.10; 95% CI: 6.50, 21.69; POC, ARD: 10.27; 95% CI: 4.70, 15.84; and Mestizo, ARD: 7.70; 95% CI: 4.32, 11.09) compared to Indigenous groups.

All five different outcomes were more frequently reported in urban compared to rural areas, with the largest difference in sexual violence (ARD: 9.27; 95% CI: 6.76, 11.79). Women from the Highlands and Amazon regions reported more often being exposed to all forms of violence except sexual, where the risk difference was smaller in the Amazon region. The highest risk difference in the Highlands was for total violence (ARD: 12.65; 95% CI: 10.22, 15.07) and in the Amazon region for psychological (ARD: 13.88; 95% CI: 11.09, 16.66) and physical (ARD: 13.49; 95% CI: 10.50, 16.48) violence.

## Discussion

This study investigated the prevalence and associated factors for VAW in Ecuador using data from a national survey among 17,211 women aged 15 years old and over conducted in 2019.

Our analysis showed a lifetime reported prevalence of 64.86% for all forms of violence which is higher than the previous national study from 2011 (60%) [17], and higher than the WHO multi-country study from 2005, where between 21 and 57% of the participating women older than 15 years old reported ever having experienced violence by any perpetrator [26]. Around one-third of the participants (32.67%) had ever experienced sexual violence, again higher reported prevalence than the previous 2011 national survey (25.7%) [17]. This reported prevalence was also higher than the lifetime prevalence of non-partner sexual violence (10.7%) in the Americas region reported by WHO in 2013 [5]. The prevalence of sexual violence by any perpetrator among women ever married or in union was lower in several Latin American countries compared to our study, including 17.9% in Bolivia, 16.6% in Colombia and 15.2% in Nicaragua [6]. In several Latin American countries, the prevalence of ever having experienced sexual IPV among women married or cohabiting were as follows: 9.1% in Nicaragua, 7.6% in Colombia, 6.5% in Peru and 5.4% in the Dominican Republic [27].

Similar figures as for sexual violence were found for physical violence (34.56%). A Latin American study reported a lifetime prevalence for this type of violence of 31% among women ever married or in union by a partner in Ecuador, whereas in the rest of the countries, numbers ranged from 17.9% in Paraguay to 52.3% in Bolivia during the period 2003 to 2006 [6]. A more recent systematic review showed a lifetime prevalence of physical IPV of 32.3% in Colombia, 30.6% in Peru, 20% in Nicaragua and 19.8% in Mexico [27].

As other IPV-related studies have shown [6, 14, 28], psychological violence was the most common type of VAW (56.92%) referred by the participating women. This figure was higher than the one previously reported in the country (53.9%) [17] and in an IPV study from Bolivia (50%) [14].

The widespread norms of machismo and old-fashioned gender roles are crucial reasons to understand why VAW is so common in Ecuador in particular and in Latin America in general [29].

Our study also showed that 16.38% of the women had been victims of economic violence. Although not so commonly explored in the literature, studies regarding IPV and VAW from Mexico (60%) [30], Bolivia (55.3%) [31] and Peru (22.2%) [32] confirm the spread of this problem. In these countries, women often have more unfavourable working conditions, make less money than men and are supposed to take care of the house and family within the relationship [33]. This makes women more vulnerable to changes in the economy and more dependent on their partner or husband for financial support, which could explain the high prevalence of this type of violence.

Literature regarding factors associated to VAW in Ecuador and Latin America is extremely scarce and focused mainly on IPV. One study from Bolivia identified several common risk factors for IPV such as urban residence, witnessing violence between parents as a child and women being employed [14]. In another study among women attending health services in Brazil, women witnessing violence as a child and mental disorders were factors associated to VAW [13]. Additionally, having a partner who abuses alcohol is a common significant risk factor for domestic violence in Latin America and globally [6, 12, 13, 34].

While sexual violence was more common in the youngest group (15–25), physical and psychological violence were more frequent in the 26–55 age group. Non-partner sexual violence in younger ages seems to be a common finding [6, 12, 13, 35] with the perpetrator typically a family member or a male known to the victim [35]. Although age was not associated with IPV in the Bolivian study [14], it played a role in a recent study from Panama [36] where younger women reported more frequently physical or verbal violence by any perpetrator during the past year. The role of age as an associated factor seems to be very contextual and related to the type of violence, with studies showing no association [14], higher risk among youth [26, 36] or among older age groups [6]. Differences in social and individual tolerance to violence or in reporting among age groups due to increase awareness have been proposed as possible explanations [35, 36].

Not being able to have an education was associated with all types of violence except sexual. These results are in line with previous research from Latin America and globally [5, 6, 13, 26]. Women who have not been able to get an education are more likely to be in a socially disadvantaged position [12] where many are unemployed and dependent on others for money, most often a male partner. This dependency alongside social norms that accept gender inequality and violence, as well as a lack of awareness of their rights, constitute obstacles for women to seek help and combat the abuse [29]. Similar to our findings of sexual violence, a study from Peru in 2005 also reported an increased risk of experiencing sexual IPV among highly educated women [37], while in the Bolivian study the risk among highly educated women was additionally higher for physical violence, especially when the woman had a higher education than her partner [14]. In countries where traditional gender roles and “machismo” are prominent, highly educated women may challenge male superiority and thus increase the risk for violence [33].

The same as in other studies, VAW was common among separated/divorced women, [6, 26] but in our study being married or living with a partner were also factors related to violence. Two possible reasons have been discussed in the literature for the higher prevalence among separated/divorced: that violence itself is a reason for ending the marriage, and that separated or divorced women are more willing to discuss their experiences with less fear of consequences [26]. In the Bolivian study, unmarried women living with a partner reported higher prevalence of experiencing psychological violence, arguing that norms for cohabiting without marriage were weaker and thus the male exerted more control over the women [14].

Sexual violence was more common among non-Indigenous compared to Indigenous populations. This result is different from previous data from Ecuador where both a higher prevalence of physical and sexual IPV was reported among Indigenous women [6]. In the study from Panama, physical or sexual IPV were more common among Indigenous [36], which contradicts our results. A Mexican study reported that societies with an Indigenous minority had higher prevalence of IPV in comparison to societies that were exclusively Indigenous, indicating that being part of a minority group can be a risk factor for VAW [38]. Reasons for why non-Indigenous women reported a higher frequency of sexual violence could be related to differences regarding socio-cultural norms within the ethnic groups. While violent attitudes could be more frequent among non-Indigenous societies, women in these groups might also be more open to report and less to tolerate violence compared to Indigenous women.

Women living in an urban setting had a significantly higher risk difference for all forms of violence, the difference being largest for sexual violence. This result has often been found in the literature on violence in Ecuador as well as in other Latin American countries [6, 14, 37]. A possible reason for the higher risk could be that rural women live in more traditional societies where violence is more often justified and hence less reported. For instance, in a study from Ecuador in 2004, 50% of the participating women living in a rural area said that “wife-beating is justified for at least one reason” compared to 31% of women in urban areas [6].

An increased risk difference for sexual and economic violence was observed among women living in the Highlands, whereas a higher risk for physical violence was observed in the Amazon region; in both regions the risk difference for psychological violence was also lager compared to the rest of the country. While some of the reasons presented above could explain these regional differences, further research would be needed to understand these variations.

### Methodological considerations

Important strengths of this study include the large study sample representative of the regions and urban/rural settings nationally. The comprehensive training received by the interviewers, and the thorough sampling procedures implemented to collect the data contribute to strengthen the internal validity of the study. The survey included multiple questions and different forms of violence (not only sexual and physical) using the definition of violence stated in the National Comprehensive Organic Law to Prevent and Eradicate Violence, which contributed to capture a complete picture of violence against women in the country. Absolute, instead of relative, differences were used as measures of association to avoid interpreting social factors of the victims as determinants of the outcomes.

However, there are several limitations that should be taken into account. Given that the data used followed a cross-sectional design, attributions to causality cannot be established. In addition, since the data were collected by face-to-face interviews and the exposure to violence investigated during their lifetime, risks for interviewer and recall bias exist, respectively. Certain social desirability bias could also not either be rejected. Due to social norms and fear of consequences, women might have also underreported violent events leading to an underestimation of the reported prevalence. Despite the broad definition of violence used in this study, the literature is clear [12, 27] regarding the challenges of defining and measuring the different types of violence, making comparisons between countries difficult. Although several social-ecological factors were included (and controlled for) in our analysis, other relevant factors for VAW could not be considered due to their non-availability in the survey.

## Conclusion

The aim of this study was to determine the prevalence of VAW and to assess its sociogeographical distribution in Ecuador. Our results showed that 64.86% of the women reported to ever having experienced some form of violence in their lifetime, most frequently psychological violence. Most types of violence were more common among the youth, those with low education, those who lived with a partner, those who were divorced/separated, or those who resided in an urban setting or in the Highlands or Amazon regions. Specifically, the risk difference for sexual violence was larger among higher educated women and those belonging to a non-Indigenous group.

VAW continues to be a huge public health problem in Ecuador. Empowering women and making them aware of their rights is essential to provide them with resources (institutional, legal, economic, political) to combat violence. It is also necessary to challenge traditional norms favouring male dominance and the widespread acceptance of violence in order to reach gender equality in the society. Although the country has taken some steps in the right direction, current policies and laws to protect women need to be expanded and effectively implemented to reduce VAW in Ecuador.

## Supplementary Information

**Additional file 1.**

## Data Availability

The data that support the findings of this study are publicly available at: https://www.ecuadorencifras.gob.ec/violencia-de-genero/

## Abbreviations

VAW: Violence against women
IPV: Intimate Partner Violence
CI: Confidence Interval
PR: Prevalence Ratio
ARD: Adjusted Risk Difference
WHO: World Health Organization
POC: People of colour
