# Supplementary material for: Prevalence and sociogeographical inequalities of violence against women in Ecuador: a cross-sectional study
Source: Int J Equity Health. 2021 Jun 2;20:130. doi: 10.1186/s12939-021-01456-9 (PMC8170937; doi:10.1186/s12939-021-01456-9)
Supplement: Supplementary file 1 — Additional file 1. [file 12939_2021_1456_MOESM1_ESM.docx]

Table S1: Characteristics of study participants and prevalence of the different outcomes (within groups), Ecuador 2019

| **Variables** |  | | **Total sample (%)** | **Total violence (%)** | **Sexual violence (%)** | **Physical violence (%)** | **Psychological violence (%)** | **Economic violence (%)** |
| --- | --- | --- | --- | --- | --- | --- | --- | --- |
|  | **Total sample** | | 17211 (100) | 11163 (64.86) | 5622 (32.67) | 6100 (35.44) | 9797 (56.92) | 2819 (16.38) |
| **Individual** |  | | | | | | | |
| **Age** | 15-25 | | 4213 (24.48) | 2432 (21.78) | 1608 (28.60) | 919 (15.06) | 1853 (18.91) | 362 (12.86) |
|  | 26-35 | | 2816 (16.36) | 1985 (17.79) | 1117 (19.87) | 1108 (18.17) | 1741 (17.77) | 493 (17.49) |
|  | 36-45 | | 3048 (17.71) | 2071 (18.56) | 961 (17.09) | 1142 (18.73) | 1885 (19.24) | 596 (21.12) |
|  | 46-55 | | 2813 (16.34) | 1856 (16.63) | 881 (15.68) | 1147 (18.80) | 1696 (17.32) | 547 (19.41) |
|  | 56-65 | | 2270 (13.19) | 1467 (13.14) | 594 (10.57) | 953 (15.62) | 1358 (13.86) | 422 (14.97) |
|  | >65 | | 2051 (11.92) | 1351 (12.10) | 461 (8.19) | 832 (13.63) | 1264 (12.90) | 499 (14.16) |
| **Education** | University or higher | | 3631 (21.10) | 2270 (20.34) | 1498 (26.65) | 984 (16.13) | 1852 (18.90) | 492 (17.46) |
|  | Secondary | | 6997 (40.65) | 4536 (40.63) | 2543 (45.23) | 2317 (37.99) | 3896 (38.77) | 1110 (39.37) |
|  | Primary/Middle | | 5699 (33.11) | 3733 (33.44) | 1411 (25.10) | 2340 (38.36) | 3455 (35.26) | 1020 (36.19) |
|  | None | | 885 (5.14) | 624 (5.59) | 170 (3.03) | 459 (7.52) | 595 (6.07) | 197 (6.98) |
| **Relationship** |  | | | | | | | |
| **Marital status** | Single | 4555 (26.47) | | 2502 (22.41) | 1687 (30.01) | 903 (14.80) | 1866 (19.04) | 250 (8.85) |
|  | Living with partner | 3562 (20.70) | | 2424 (21.72) | 1119 (19.91) | 1396 (22.88) | 2208 (22.53) | 527 (18.70) |
|  | Married | 5524 (32.10) | | 3494 (31.30) | 1473 (26.19) | 1936 (31.75) | 3170 (32.35) | 664 (23.55) |
|  | Separated/divorced | 2474 (14.37) | | 2073 (18.57) | 1118 (19.88) | 1397 (22.90) | 1920 (19.59) | 1163 (41.26) |
|  | Widowed | 1097 (6.37) | | 670 (6.00) | 225 (4.01) | 468 (7.67) | 634 (6.48) | 216 (7.64) |
| **Societal** |  | | | | | | | |
| **Ethnicity** | Indigenous | 1237 (7.19) | | 793 (7.10) | 244 (4.34) | 550 (9.01) | 728 (7.43) | 201 (7.14) |
|  | POC | 1204 (7.00) | | 780 (6.99) | 389 (6.93) | 450 (7.38) | 711 (7.26) | 216 (7.66) |
|  | Mestizo | 14135 (82.13) | | 9199 (82.41) | 4741 (84.32) | 4905 (80.40) | 8022 (81.88) | 2318 (82.19) |
|  | White and others | 635 (3.69) | | 391 (3.50) | 248 (4.41) | 196 (3.20) | 336 (3.43) | 85 (3.01) |
| **Area** | Rural | 4936 (28.68) | | 3098 (27.75) | 1131 (20.11) | 1883 (30.87) | 2832 (28.91) | 733 (26.01) |
|  | Urban | 12275 (71.32) | | 8065 (72.25) | 4492 (79.89) | 4217 (69.13) | 6965 (71.09) | 2086 (73.99) |
| **Regions** | Coast | 8382 (48.70) | | 5022 (44.99) | 2620 (46.60) | 2603 (42.66) | 4374 (44.65) | 1226 (43.48) |
|  | Highlands | 7984 (46.39) | | 5555 (49.76) | 2557 (49.03) | 3132 (51.34) | 4887 (49.89) | 1448 (51.36) |
|  | Amazon | 789 (4.58) | | 556 (4.98) | 233 (4.14) | 349 (5.72) | 510 (5.20) | 141 (5.00) |
|  | Galapagos & Others | 56 (0.33) | | 30 (0.27) | 13 (0.23) | 17 (0.28) | 26 (0.26) | 5 (0.16) |
